# Clinicopathological Characteristics of Patients with Non-Small-Cell Lung Cancer Who Harbor *EML4-ALK* Fusion Gene: A Meta-Analysis

**DOI:** 10.1371/journal.pone.0117333

**Published:** 2015-02-23

**Authors:** Fengzhi Zhao, Meng Xu, Honcho Lei, Ziqi Zhou, Liang Wang, Ping Li, Jianfu Zhao, Penghui Hu

**Affiliations:** Department of Oncology, the First Affiliated Hospital, Jinan University, Guangzhou, China; University of Algarve, PORTUGAL

## Abstract

**Background:**

A novel fusion gene of echinoderm microtubule-associated protein-like 4 (*EML4*) and anaplastic lymphoma kinase (*ALK*) has been recently identified in non-small-cell lung cancers (NSCLCs). Patients with the *EML4-ALK* fusion gene demonstrate unique clinicopathological and physiological characteristics. Here we present a meta-analysis of large-scale studies to evaluate the clinicopathological characteristics of NSCLC patients harboring the *EML4-ALK* fusion gene.

**Methods:**

Both English and Chinese databases were systematically used to search the materials of the clinicopathological characteristics of patients with NSCLC harboring the *EML4-ALK* fusion gene. Pooled relative risk (RR) estimates and the 95% confidence intervals (95% CI) were calculated with the fixed or random effect model. Publication bias and chi-square test were also calculated.

**Results:**

27 retrospective studies were included in our meta-analysis. These studies included a total of 6950 patients. The incidence rate of *EML4-ALK* fusion in NSCLC patients was found to be 6.8% (472/6950). The correlation of the *EML4-ALK* fusion gene and clinicopathological characteristics of NSCLC patients demonstrated a significant difference in smoking status, histological types, stage, and ethnic characteristics. The positive rate of the *EML4-ALK* fusion gene expression in females were slightly higher than that in males, but not significantly (*P* = 0.52). In addition, the *EML4-ALK* fusion gene was mutually exclusive of the *EGFR* and *KRAS* mutation genes (*P* = 0.00).

**Conclusion:**

Our pooled analysis revealed that the *EML4-ALK* fusion gene was observed predominantly in adenocarcinoma, non-smoking and NSCLC patients, especially those diagnosed in the advanced clinical stage of NSCLC. Additionally, the *EML4-ALK* fusion gene was exclusive of the *EGFR* and *KRAS* mutation genes. We surmise that IHC assay is a valuable tool for the prescreening of patients with *ALK* fusion gene in clinical practice, and FISH assay can be performed as a confirmation method. These insights might be helpful in guiding the appropriate molecular target therapy for NSCLC.

## Introduction

Lung cancer is the most prevalent cancer and leading cause of cancer-related deaths worldwide [1]. Despite improvements in therapeutic methodology, including surgery, chemotherapy and radiotherapy, the average prognosis of lung carcinoma still remains unsatisfactory and the five year survival rate is merely 15% [2]. Among those lung cancer patients, non-small cell lung cancer (NSCLC) accounted for approximately 80%-85% of lung cancer cases [3]. Conventionally, the clinical pathological stage is the important system for predicting the survival rate in patients [4]; the recent discovery of novel molecular signal alterations also may be involved in defining a therapy, which may turn out to be more effective and with less side effects than conventional treatment.

Increased attention has been garnered in the development and application of drugs that target specific molecules which expressed on NSCLC cells and great success has been reported in NSCLC patient study groups [5,6]. These methods include signaling transduction and angiogenesis inhibitors, such as the epidermal growth factor receptor (*EGFR*) targeted drugs [7]. Therefore, the identification of the key ontogeny for cancer was a critical step in developing molecular-targeting agents.

In the year 2007, the fusion of echinoderm microtubule-associated protein-like 4 (*EML4*) genes with anaplastic lymphoma kinase (*ALK*) was found in lung cancer [8]. The fusion of the N-terminal half of *EML4* and the intracellular kinase domain of *ALK* within chromosome 2p lead to expression of chimeric tyrosine kinase [9]. The *EML4-ALK* fusion gene possessed potent critical biological activity in vitro and in vivo, such as cell proliferation, apoptosis and metastasis [10], which can be effectively blocked by the *ALK* kinase inhibitor (Crizotinib) [11], which lends a supporting role for the *EML4-ALK* fusion gene in lung tumorigenesis. To identify patients likely to benefit from Crizotinib, it is necessary to develop a robust and effective diagnostic algorithm to detect the *EML4-ALK* fusion gene when screening patients for treatment with Crizotinib. Currently, the following three methodologies are used to detect the *EML4-AL*K fusion gene, which include: fluorescence in situ hybridization (FISH), reverse transcriptase-polymerase chain reaction (RT-PCR) and immunohistochemical (IHC). However, the best algorithm for screening the *EML4-ALK* fusion gene in clinical lung cancer populations remains to be determined, since the three methodologies described above have different advantages and disadvantages. To improve the detection efficiency of the three methodologies, we investigated if combining the clinicopathological characteristics of NSCLC with the *EML4-ALK* fusion gene would yield useful information for the effective pre-screening of patients with the *EML4-ALK* fusion gene in clinical practice.

Despite a large number of studies on patients harboring the *EML4-ALK* fusion gene demonstrating unique clinical physiological and pathological characteristics [12], detailed clinicopathological profiles remain unclear because of the small number of cases identified. To correlate the *EML4-ALK* fusion gene with the NSCLC profile (including smoking status, gender, tumor types, stage and ethnic characteristics) and ascertain the relationship of *EML4-ALK* with *EGFR* and *KRAS* mutations, we performed the present meta-analysis of 6950 patients from 27 studies.

## Methods

### Search Strategy

Electronic searches were performed until April 2014 and included various sources, such as MEDLINE, Embase Databases, Elsevier Science Direct, ISI Web of Science, China National Knowledge Internet, China Biology Medical Literature Database, and the Database of Chinese Scientific and Technical Periodicals. No language restrictions were applied. The keywords were as follows: “non-small cell lung cancer or NSCLC”, “echinoderm microtubule-associated protein-like 4 or *EML4*”, “anaplastic lymphoma kinase or *ALK*”, “fusion gene”, “physiological and pathological characteristics”. We searched the reference lists of relevant reviews, editorials, studies, meeting abstracts and letters. We used the Sciences Citation Index to cross reference for further studies that fulfilled the eligibility criteria.

### Study selection

The studies included in this meta-analysis according to our predetermined criteria are as follows: (1) the trials that include the full text of the paper published in peer-reviewed English and Chinese journals or reports of presentations at major oncology meetings; (2) evaluation of the associations between the *EML4-ALK* fusion gene and clinicopathological characteristics in NSCLC patients; (3) similarity in the patients’baseline characteristics.

### Data extraction and quality assessment

Two reviewers (Zhao and Lei) independently collected the data with the standard protocol. The following criteria were set to screen the articles which were eligible for our study: (1) expression of the *EML4-ALK* fusion gene was evaluated in primary lung cancer tissue as opposed to metastatic tissue; (2) methods used to detect the *EML4-ALK* fusion gene expression, including IHC, FISH or RT-PCR; (3) the histological type of the tumors was NSCLC; (4) comparison of the risk ratio (RR) and its confidence interval (CI) between patients who harbor the *EML4-ALK* fusion gene over expression and the counterparts were described or statistically extractable from the data in the article; (5) when multiple articles were published by the same authors or groups, the most informative or newest single article was selected. The studies were evaluated with the Downs and Black quality assessment method[13]; (6) potential disagreements were resolved by discussion and consensus with senior investigator (Xu).

### Statistical and Sensitivity Analysis

This meta-analysis was performed in the RevMan 5.2 software. Statistical calculations were used with SPSS (version 17.0 SPSS Inc., IL, USA). The relative risk (RR) and the mean difference with 95% confidence intervals (95% CI) were calculated for the continuous outcomes and dichotomous outcomes, respectively. P<0.05 was considered as a significant difference in the value between the two groups. The I^2^ statistic was used to investigate the heterogeneity among the studies. The heterogeneity was explored by I^2^ and χ^2^, I^2^<50% indicated a small inconsistency and I^2^>50% indicated a large inconsistency. When there was a statistical difference in terms of the heterogeneity (I^2^>50%), the random-effect model was used to pool the data; Otherwise, a fixed-effect model was selected.

### Publication bias

For publication bias estimating, we can visually observe any significant statistically symmetrical differences, according to the funnel plot.

## Results

### Description of the Studies

**Fig 1:**
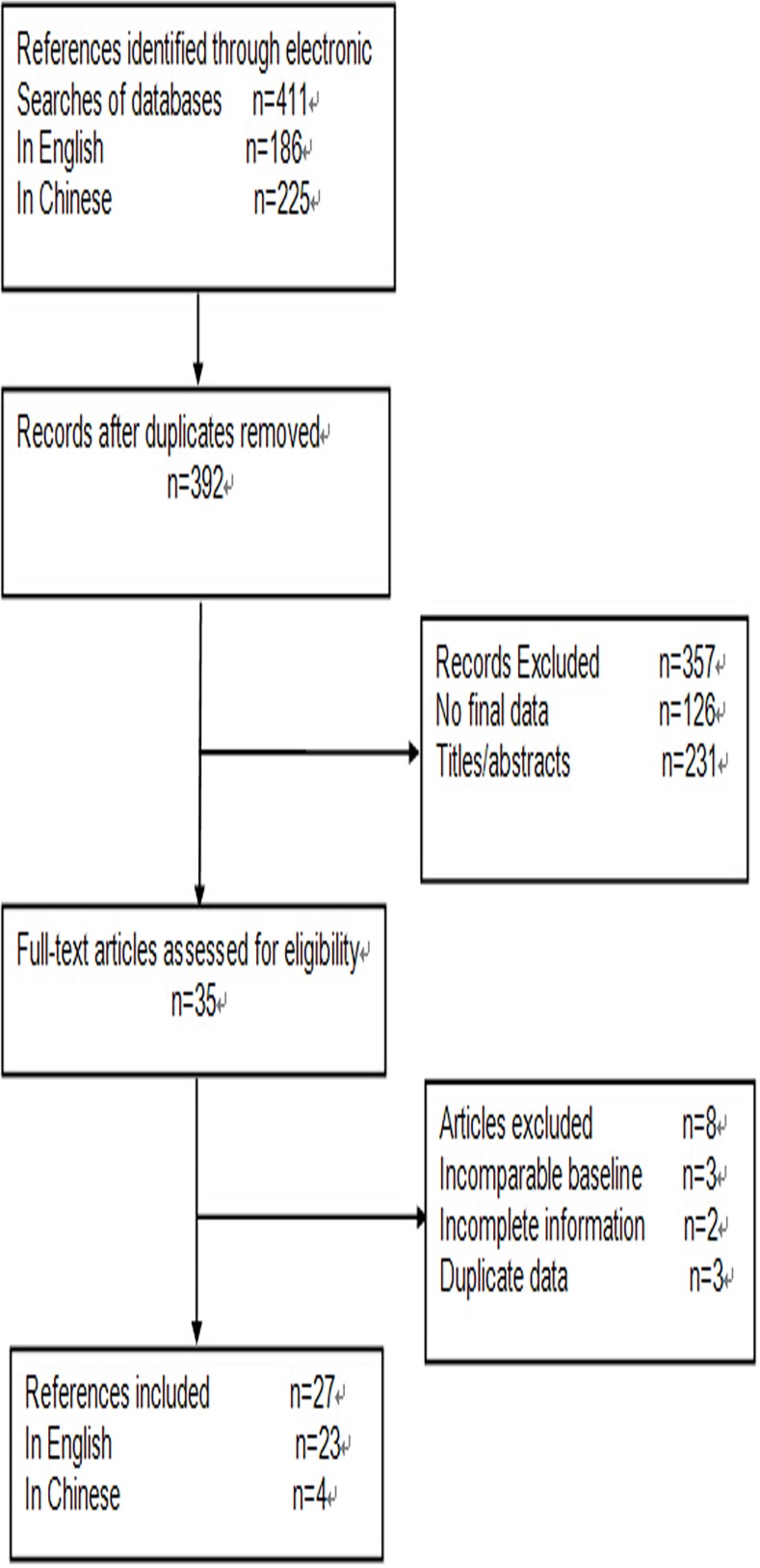
Flow diagram of study selection.

27 retrospective cohort studies coincided with our criteria and are included in this meta-analysis. A total of 6950 patients were included in the 27 studies, among which 24 (5130 cases) estimated the association of fusion of the *EML4-ALK* gene in NSCLC with a history of smoking; while 17 studies emphasized the association of the *EML4-ALK* rearrangement to tissue types (3360 cases); 13 papers reflected the relation of the *EML4-ALK* fusion gene to clinical stages (2876 cases) and 26 researches showed the association of this fusion gene and the gender of patients (5797 cases). To search algorithm, the results of the selection criteria and search strategies are shown in Fig. 1, and the characteristics of patients and detected methods of the *EML4-ALK* fusion gene are shown in Table 1.

**Table 1:** Characteristics of included studies regarding patients and detected methods.

**Author(ref.)**	**Year**	**Total**	**Gender**		**Smoking**		**Pathological Type**		**TMN(stage)**				**methods**
			**M**	**F**	**(-)**	**(+)**	**AD**	**NAD**	**I**	**II**	**III**	**IV**	
Inamura K [14]	2008	221	80	69	65	84	149	72	63	85a	-	-	RT-PCR
Soda M[15]	2007	75	22	11	9	24	18	15	-	-	-	-	RT-PCR
Shinmura K[16]	2008	77	39	38	41	22	50	27	-	-	-	-	RT-PCR
Kentaro I[17]	2008	253	134	119	142	110	-	-	-	-	-	-	RT-PCR
Shaw AT [18]	2009	141	48	93	59	82	89	52	25	1	9	96	FISH
Wong DW [19]	2009	266	132	134	141	125	209	57	153	47	60	6	RT-PCR
Inamura K [20]	2009	363	134	119	105	147	253	110	143	110a	-	-	RT-PCR, FISH
Martelli MP [21]	2009	120	96	24	16	101	63	57	65	21	22	11	RT-PCR
Rodig SJ[22]	2009	358	138	220	85	243	358	0	169	29	67	93	FISH, IHC
Jokoji R [23]	2010	254	130	124	51	84	-	-	-	-	-	-	IHC
TakahashiT [24]	2010	313	111	100	92	119	211	102	141	20	42	8	RT-PCR
Zhang X [25]	2010	103	74	29	52	51	62	41	63	18	20	2	RT-PCR
Sanders HR [26]	2011	55	-	-	-	-	37	18	-	-	-	-	RT-PCR
Shaw AT [27]	2011	411	177	234	175	237	377	35	-	-	-	-	FISH
Sequist LV [28]	2011	546	228	318	128	415	440	106	165	32	105	241	RT-PCR
Jin G [29]	2012	167	85	82	73	94	121	46	93	74	-	-	RT-PCR
Kim HR [30]	2012	229	30	199	-	-	215	14	43	31	61	94	FISH,
Koivunen JP [31]	2012	305	187	204	69	184	208	97	183	59	50	9	RT-PCR
Lin XM [32]	2012	102	54	48	73	29	73	29	34	17	40	11	RT-PCR
Han XH[33]	2013	137	56	81	107	32	135	4	-	-	27	112	RT-PCR, FISH, IHC
Takamochi k [34]	2013	222	117	105	101	120	-	-	150	71a	-	-	RT-PCR, FISH, IHC
Zhang YG [35]	2013	473	314	159	180	293	341	132	166	209b	-	98	RT-PCR, FISH, IHC
Fang P[36]	2013	60	34	26	35	25	-	-	16	16	18	10	FISH
Zhong S [37]	2013	268	183	85	118	123	132	79	-	-	-	-	RT-PCR
Wang M [38]	2013	245	178	67	118	127	114	131	62	113	40	30	IHC
Li Y[39]	2013	208	147	61	78	130	95	113	49	43	106	10	RT-PCR
Yang JJ[40]	2014	977	182	212	308	86	377	17	75c	-	319d	-	RT-PCR, FISH, IHC
Total		6950	3238	2734	2035	2871	3655	1224	1734	404	561	821	-

M:male; F:female AD: adenocarcinom; NAD: non-adenocarcinoma; a.Patients of stage II-IV; b. Patients of stage II-IIIA; c. Patients of stage I-II; d. Patients of stage III-IV; RT-PCR: real-time polymerase chain reaction; IHC: immunohistochemestry; FISH: fluorescence in situ hybridization.

### Direct meta-analysis and pooled outcomes

**Fig 2:**
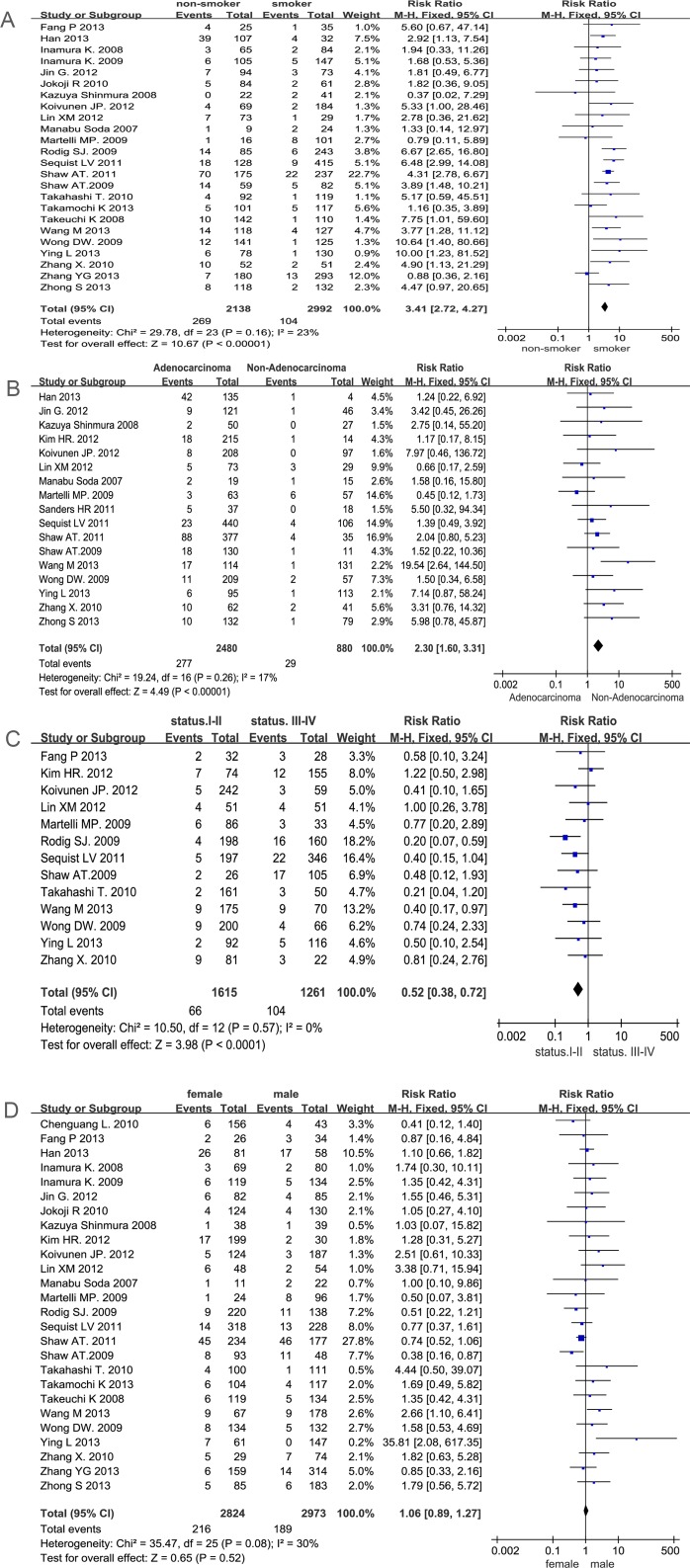
Meta-analysis of data for EML4-ALK. (A smokers vs no-smokers; B adenocarcinomas vs non-adenocarcinomas; C stages I-II vs stages III-IV; D male vs female). Forest plot of the Relative Risk (RR) of the clinicopathological characteristics with *EML4-ALK* fusion gene patients. The RR estimate of each individual trial corresponds to the middle of the squares and the horizontal line gives the 95% CI. On each line, the numbers of events are represented as fractions of the total number; random choices are shown for both treatment groups. For each subgroup, the sum of the statistics, along with the summary RR are represented by the middle of the solid diamonds. A test of heterogeneity between the trials within a subgroup is given below in summary of the statistics.

Meta-analysis of the literature revealed 27 publications, which included 6950 NSCLC patients; 472 of these patients (6.8%) harbored the *EML4-ALK* fusion gene. 24 studies out of 27 documented the correlation between smoking history and the *EML4-ALK* fusion gene. We detected no significant bias between the two groups (P = 0.16 I^2^ = 23%) when the fixed effects model was used. The combined result is shown in Fig. 2A. Compared with smoking cases, non-smokers with NSCLC have a statistically significant higher risk in the presence of the *EML4-ALK* fusion gene (12.6% vs 3.4%, RR = 3.41, 95%CI, 2.72–4.27, *P*<0.01). 17 studies assessed the *EML4-ALK* fusion gene in adenocarcinoma and non-adenocarcinoma groups, and heterogeneity was indentified through the 16 reports (*P* = 0.26 I^2^ = 17%). Then data were analyzed using a fixed effects model. The results indicated that the *EML4-ALK* fusion frequency is higher in the adenocarcinoma group than in the non-adenocarcinoma group (11.2% vs 3.3%, RR = 2.30, 95%CI, 1.60–3.31, *P*<0.01) in Fig. 2B. 13 studies expressed the association between tumor stage and the *EML4-ALK* fusion gene. There is no significant bias between stage I-II and stage III-IV (*P* = 0.57 I^2^ = 0%); therefore, data were analyzed using a fixed effects model. Our results suggest that there was a statistically significant increase in the frequency of *EML4-ALK* mutations in stage III-IV than in stage I-II (8.2% vs 4.0%, RR = 0.52, 95%CI, 0.38–0.72, *P*<0.01) in Fig. 2C. In addition, 26 out of these 27 studies documented the *EML4-ALK* fusion in female and male groups. We detected no significant bias between the two groups (*P* = 0.08 *I^2^* = 30%) and analyzed the data using a fixed effects model. Our results suggest that there was no significant difference between the male and female groups. (7.6% versus 6.3%, RR = 1.06, 95%CI, 0.89–1.27, p = 0.52) in Fig. 2D.

We also extended our study to perform three methods (FISH, RT-PCR and IHC) for direct comparison of sensitivity and specificity. Among the 3,813 patients included for the FISH study, 336 patients (8.8%,) were found to have the *EML4-ALK* rearrangement. Among the 5,236 patients, 280 (5.3%) were significantly positive by detection of RT-PCR. A total of 2,688 patients had successful IHC staining detection of the *EML4-ALK* fusion expression and 191 patients harbored the *EML4-ALK* fusion gene (7.1%) in Table 2.

**Table 2:** The detecting methods of the *EML4-ALK* fusion gen.

**Methods**	***ELM4-ALK* fusion**		**Total**
	**positive**	**negative**	
FISH	336(8.8%)	3,477(91.2%)	3,813
RT-PCR	280(5.3%)	4,956(94.7%)	5,236
IHC	191(7.1%)	2,477(92.9%)	2,668

The χ2 test indicate there was significant difference among the three diversified methods in the diagnostic detection rate of EML4-ALK fusion gene (χ2 = 21.04, p = 0.000).

In this analysis, 27 studies evaluated the *EML4-ALK* fusion gene in different ethnicity groups. The difference of fusion rates in Asian and non-Asian population was significant; there were 6,950 patients selected from 27 randomized trials, 4906 in Asian and 2,044 from non-Asian populations. Patients in non-Asian ethnicity groups had a higher mutation rate than those in Asian ethnicity groups (8.5% versus 6.1% χ^2^ = 12.80 P = 0.00) in Table 3.

**Table 3:** Comparison of EML4-ALK mutation rate between Asian and non-Asian.

**Group**	***EML4-ALK* fusion**		**Total**
	**positive**	**Negative**	
Asian	299(6.1%)	4,607(93.9%)	4,906
non- Asian	173(8.5%)	1,871(91.5%)	2,044
Total	472(6.8%)	6,478(93.2%)	6,950

The χ2 test indicated that the rate of ALK mutation in non-Asian group was statistically higher than the Asian group (χ2 = 12.8, p = 0.000).

There were 694 patients with *EGFR* mutations and 107 patients with *KRAS* mutations. Additionally, there were 214 patients with the *EML4-ALK* fusion gene, as summarized in Table 4. 199 patients who harbor the *EML4-ALK* fusion gene had wild-type *EGFR* and *KRAS*. Statistical analysis demonstrated a significant association of the *EML4-ALK* fusion gene with wild-type *EGFR* (*P* = 0.00 McNemar test) and *KRAS* (*P* = 0.00 McNemar test). Nevertheless, we identified 15 *EML4-ALK* fusion NSCLC patients in our study that showed coexistent mutations in *EGFR* in Table 4.

**Table 4:** The correlation with ALK fusion mutation and EGFR/KRAS mutation.

**Group**		**EGFR**		**Total**	**KRAS**		**Total**
		**(+)**	**(-)**		**(+)**	**(-)**	
EML4-ALK	(+)	15(2.1%)	146(97.9%)	161	0(0%)	53 (100%)	53
	(-)	679(12.1%)	1,059(87.9%)	1,738	107(8.3%)	588(91.7%)	695

The McNemar test illustrated that there was a statistically significant difference in the case number between *EML4-ALK* fusion and *EGFR* mutation (p = 0.000), the outcome of *KRAS* followed the same pattern (p = 0.000).

### Publication Bias

**Fig 3:**
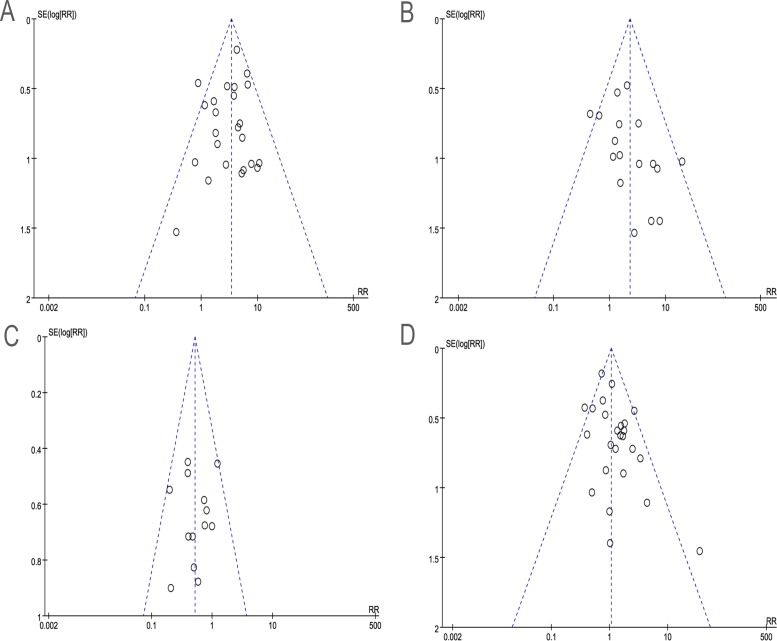
Funnel plot of the outcome of clinicopathological characteristics and the EML4-ALK fusion gene. (smokers vs non-smokers; B adenocarcinomas vs non-adenocarcinomas; C stages I-II vs stages III-IV; D male vs female).

Publication bias can exist when non-significant findings remain unpublished. Begg’s funnel plot was performed to assess the potential publication bias in all literature. As shown in Fig. 3, the symmetric shape of funnel plots does not reveal any evidence of publication bias.

## Discussion

According to tumor-specific biological characteristics, molecularly targeted therapies recently showed a new strategy that demonstrated the importance of small subgroup patients. The *EML4-ALK* fusion gene represents a new subgroup of NSCLC patients who respond positively to ALK inhibitors [11].

Presently, the most comprehensive meta-analysis regarding the clinical characteristics of the *EML4-ALK* fusion gene was done in our study. We fully analyzed 6,950 cases from 27 articles. Our findings showed a low incidence (6.8%) of the *EML4-ALK* translocation among unselected NSCLC patients; this proved consistent with previous reports (1.4%~11.6%) [16,17,20]. Since the incidence of *EML4-ALK* is low in NSCLC patients, it is necessary to elucidate clinicopathological characteristics of the *EML4-ALK* fusion gene-positive lung cancer to improve screening efficiency. Our results indicated that the *EML4-ALK* fusion gene occurred predominantly in non-smoking, adenocarcinoma patients, although no statistical difference was found between male and female patients. The likely interpretation of this phenomenon is that adenocarcinoma account for a major portion of the female patients who seldom smoke. The findings above showed that *EML4-ALK* fusion gene-related carcinogenesis might be different from chronic inflammation induced by smoking or tuberculosis [41]. Therefore, we believe that clinical characteristics, such as smoking status, and adenocarcinoma, should be used to select patients for *EML4-ALK* fusion gene screening.

Meanwhile, we found that the incidence of EML4-ALK fusion III-IV patients was slightly higher than that in stage I-II patients. We suggest that the NSCLC patients should be finished *EML4-ALK* fusion detection before ALK molecular inhibitor treatment. In addition, this unbalanced stage distribution could have been due to the availability of fresh frozen tissues for RT-PCR, which is more likely in operable patients (stage I and III), but difficult for stage II and IV patients. However, the incidences of *EML4-ALK* rearrangements in stage III patients were higher than in the other stages [25,42,43]. In Takamochi’s study, the proportion of lymph node involvement in *EML4-ALK* fusion gene-positive adenocarcinoma was significantly more frequent than in the negative counterpart [34]. According to Paik’s report, *EML4-ALK* fusion gene-positive adenocarcinomas may metastasize to lymph nodes [44], and Vincent reported that *EML4-ALK* fusion gene-positive tumors tend to have lymph node and brain metastases [45]. All findings stated above were potentially involved in determining why the fusion gene was more frequently seen in the advanced NSCLC.

Currently, several methodologies are used to detect *EML4-ALK* fusion, including FISH, RT-PCR and IHC. In our meta-analysis, the positive rate of FISH, RT-PCR and IHC was 8.8%, 5.3% and 7.1% respectively. An accurate and reliable method for the detection of *EML4-ALK* fusion is crucial for selecting NSCLC patients who are candidates for treatment with *ALK* inhibitors. Although FISH assay has been used to identify patients with the *EML4-ALK* fusion gene in clinical trials, a gold standard method to determine the *EML4-ALK* fusion gene has not been established. Our investigation revealed that the popular methods used to detect the *EML4-ALK* fusion gene are FISH and RT-PCR. Theoretically, RT-PCR and FISH are two approaches for detecting genes fusion; however, both have considerable limitations in clinical practice. RT-PCR required fresh tissue samples for RNA extraction and a reliable FISH assay required a good fluorescence scope and technical expertise. IHC for testing *EML4-ALK* fusion is a well-established method, particularly since the cost of IHC is much lower than that of FISH. So IHC could be a much more convenient and cost-effective screening method for the *EML4-ALK* fusion gene in NSCLC patients [41]. However, because RT-PCR methodology may not identify novel rearrangements involving previously uncharacterized EML4-ALK variants or unknown fusion partners and its process may be readily contaminated, its sensitivity and specificity remain to be validated [35]. IHC has the strengths of being widely available, relatively easy to perform and retains morphological information, which allows confident assessment of aberrant genes in tumor cells. Several ALK antibodies, reported in recent studies, shows that IHC has high concordance with FISH. Thus, these results suggested that in routine practice, IHC assay is a tool of value for the prescreening of patients with ALK fusion gene in clinical practice, and FISH assay can be performed as a confirmation method. This is consistent with previous reports [34,35,46].

It should be noted, that for first time in our studies, we determined that the *EML4-ALK* fusion gene appeared more frequently in non-Asian patients, as opposed to their Asian counterparts; this means that the prevalence of *EML4-ALK* fusion in the non-Asian population is higher than that in other ethnicities. This result indicates that the *EML4-ALK* fusion may be linked to non-Asian ethnicity, as opposed to *EGFR* mutations, which are linked to Asians. Wu recently reported that among NSCLC patients with available date on ethnicity and variant type data for the *EML4-ALK* fusion gene, variant 3(52.3%) was the most common type in the Chinese population, while variant 1(75.7%) was most common in the Caucasian population [47]. These results further indicated that the prevalence of the *EML4-ALK* fusion gene may vary amongst different ethnic groups.

Although *EML4-ALK* fusion and *EGFR* mutations were previously reported to be mutually exclusive, several studies have shown that *EML4-ALK* rearrangements can occur concurrently with *EGFR* mutations [48,49]; however, these may be rare events. Our data demonstrated that there are 15(15/6950) patients who harbored concomitant *EML4-ALK* fusion and *EGFR* mutations. In addition, comparison of *EML4-ALK* rearrangement with the *KRAS* mutations in the same NSCLC samples revealed that the *EML4-ALK* fusion gene was mutually exclusive of the *KRAS* mutations. Therefore, a stepwise mode to select for gene mutations in NSCLC is suggested: first for *KRAS*, second for *EGFR*, *EML4-ALK* translocation, and then for concomitant *EML4-ALK* fusion and *EGFR* mutations. If a patient is positive for a *KRAS* mutation, no further molecular testing will be required. The treatment approach will focus on chemotherapy, as tumors with somatic mutations in *KRAS*, which encodes a GTPase downstream of *EGFR*, exhibit greater resistance to the targeted drugs. If the patient is negative for *KRAS* mutations, it will be necessary to screen for *EGFR* mutations and the *EML4-ALK* fusion gene. A positive result for either will indicate molecular treatment using *EGFR* tyrosine kinase inhibitors (TKI) or *ALK* inhibitor. When patients harbor concomitant *EML4-ALK* fusion and *EGFR* mutations, treatment strategies may be helpful, since this subgroup has a specific genotype with dual therapeutical targets. As Yang’s report [40], testing of the relative phosphorylation levels of *EML4-ALK* and *EGFR* might help to guide the selection of *ALK* inhibitor or *EGFR*-TKIs in clinical practice.

## Conclusion

Our analysis indicated that *EML4-ALK*-positive NSCLC comprised a unique subgroup of adenocarcinomas with distinct clinicopathological characteristics. We also concluded that *EML4-ALK* fusion was mutually exclusive of EGFR mutation *KRAS* mutations. Compared with non-*EML4-ALK*-positive NSCLC, this group is significantly enriched for non-smoking patients with adenocarcinoma. The positive rate of the *EML4-ALK* fusion gene expression in females was slightly higher than that in males, but not significantly. These patients typically present in late stages, which is not amenable for surgical resection. Therefore, the molecular target regimens that target the *EML4-ALK* fusion protein would be an effective, novel therapeutic method for them. Our studies represent *EML4-ALK* fusion based on the unique clinicopathological characteristics. In addition, HC assay is a tool of value for the prescreening of patients with ALK fusion gene in clinical practice, and FISH assay can be performed as a confirmation method.

## Supporting Information

S1 PRISMA Checklist (DOC)
